# The Role of SINE-VNTR-Alu (SVA) Retrotransposons in Shaping the Human Genome

**DOI:** 10.3390/ijms20235977

**Published:** 2019-11-27

**Authors:** Olympia Gianfrancesco, Bethany Geary, Abigail L. Savage, Kimberley J. Billingsley, Vivien J. Bubb, John P. Quinn

**Affiliations:** 1 Department of Molecular and Clinical Pharmacology, Institute of Translational Medicine, University of Liverpool, Liverpool L69 3GE, UK; olympia.gianfrancesco@igmm.ed.ac.uk (O.G.); abigail.savage@liv.ac.uk (A.L.S.); kimberley.billingsley@nih.gov (K.J.B.); jillbubb@liv.ac.uk (V.J.B.); 2 MRC Human Genetics Unit, Institute of Genetics and Molecular Medicine, University of Edinburgh, Edinburgh EH4 2XU, UK; 3 Division of Molecular and Clinical Cancer Sciences, Faculty of Biology, Medicine and Health, University of Manchester, Manchester M13 9PL, UK; bethany.geary@manchester.ac.uk

**Keywords:** retrotransposon, SVA, polymorphism, primate, zinc finger protein, ZNF, evolution

## Abstract

Retrotransposons can alter the regulation of genes both transcriptionally and post-transcriptionally, through mechanisms such as binding transcription factors and alternative splicing of transcripts. SINE-VNTR-Alu (SVA) retrotransposons are the most recently evolved class of retrotransposable elements, found solely in primates, including humans. SVAs are preferentially found at genic, high GC loci, and have been termed “mobile CpG islands”. We hypothesise that the ability of SVAs to mobilise, and their non-random distribution across the genome, may result in differential regulation of certain pathways. We analysed SVA distribution patterns across the human reference genome and identified over-representation of SVAs at zinc finger gene clusters. Zinc finger proteins are able to bind to and repress SVA function through transcriptional and epigenetic mechanisms, and the interplay between SVAs and zinc fingers has been proposed as a major feature of genome evolution. We describe observations relating to the clustering patterns of both reference SVAs and polymorphic SVA insertions at zinc finger gene loci, suggesting that the evolution of this network may be ongoing in humans. Further, we propose a mechanism to direct future research and validation efforts, in which the interplay between zinc fingers and their epigenetic modulation of SVAs may regulate a network of zinc finger genes, with the potential for wider transcriptional consequences.

## 1. Introduction

Retrotransposons are known drivers of genetic diversity, with the ability to mobilise and insert new copies of themselves into the genome, where they have the potential to regulate the expression of genes at that loci by altering patterns of methylation, chromatin structure, transcription factor binding, or gene splicing [1,2]. SINE-VNTR-Alus (SVAs) are the youngest and smallest family of retrotransposons and are specific to hominids, with approximately 2700 elements in the human reference genome (hg19) [3]. These elements are therefore of interest when investigating the most recent evolutionary differences between humans and other primate species, or indeed within the human population. The oldest of the seven human SVA subfamilies (SVAs A-F1), SVA A, is approximately 13.6 million years old, with the younger SVA F subfamily being around 3.2 million years old [3]. SVA subfamilies A, B, and C are found in multiple primate species, whereas the SVA E, F, and F1 subfamilies are human-specific. SVA Ds are either human-specific or found in higher primate species including gorillas, bonobos, and common chimpanzees. In this study, we therefore considered SVA A, B, and C as the evolutionary ‘older’ grouping, while SVA D, E, F, and F1 were thought of as the more recent grouping.

Human-specific retrotransposon insertions are considered to be one of the two key driving forces in the evolution of human-specific regulatory networks [4], with the second being recombination-based exaptation of highly conserved segments of ancestral regulatory DNA. This evolution of more complex gene regulatory mechanisms in higher primates and humans is likely to have in part impacted on species- and tissue-specific gene regulation and allowed greater diversity with regard to epigenetic modulation and response to environmental changes [5]. Work by Bennett et al. found that the average person harboured 56 polymorphic SVAs in their genome [6], which would be expected to contribute substantially to individual genetic variation. Bennett et al. also demonstrated that 79% of SVAs in humans are missing from their equivalent genomic sites in the chimpanzee genome, suggesting that SVAs have played a recent evolutionary role after the split between human and chimpanzee species [6,7,8,9].

Many transposable element families are known to be distributed non-randomly across the human genome. This was highlighted in the initial draft of the human genome, in which sections of the X chromosome were found to contain extremely high densities of the LINE-1 retrotransposon sequence, and *Alu* elements were found to be enriched on chromosome 19 [10]. The latter was predicted to be due to the high gene and GC content of this chromosome. We and others have similarly demonstrated that SVAs are not distributed randomly across the genome, with their distribution displaying preference for regions of high GC content [3], particularly around genic regions, with 60% of SVA elements residing either within genes or within 10 kb upstream [11]. This is consistent with reports from both Wang et al. and Tang et al. that highlight chromosome 19 as a region of the genome particularly rich in SVAs [3,12], with Grimwood et al. demonstrating that chromosome 19 has a higher proportion of transposable element-derived sequence compared to the total genome (55% vs. 44.8%) [13].

We have previously demonstrated that SVA elements are over-represented in known Parkinson’s disease gene loci [14], and are found at gene loci that modulate central nervous system (CNS) pathways [15]. Previous studies have demonstrated the ability of SVA elements to modulate gene expression both in vitro and in vivo, in human neuroblastoma cell lines and chick embryo models [11,16,17,18], which would suggest that SVA elements have the potential to regulate gene expression both through their regulatory characteristics and their location within the genome. Indeed, Kim and Hahn have highlighted the ability of SVA insertions to act as novel promoters at their site of integration, driving the expression of new, human-specific transcripts [7,8], with similar work by Kwon et al. demonstrating the inclusion of SVA sequence in numerous human transcripts [9]. More recent work by Tang et al. demonstrated that human-specific retrotransposon insertions have added 14.2 Mbp to the human genome, contributing 84 kb of expressed human-specific transcripts [12]. In this analysis, Tang et al. also found that 12.5% of human-specific SVAs overlapped with ENCODE ChIP-seq data, suggesting that their presence within our genome has provided an additional 504 transcription factor binding sites [12].

**Figure 1:**
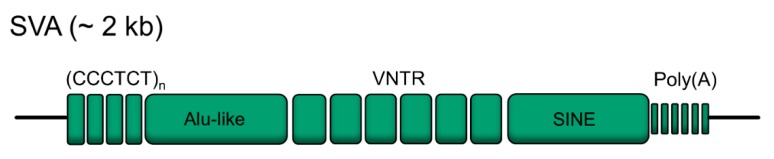
Canonical SINE-VNTR-Alu (SVA) structure. Canonical SVAs typically contain five distinct regions; a (CCCTCT)_n_ hexamer repeat at the 5′ end, an *Alu*-like domain, a variable number tandem repeat (VNTR), a SINE-derived region, and a poly(A) tail. The SVA F1 subfamily deviates from this typical structure as the (CCCTCT)_n_ hexamer has been replaced by a 5′ transduction of the first exon of the *MAST2* gene. While SVAs are approximately 2 kb in length, their size can vary due to changes in copy number in their repetitive domains.

A canonical SVA is comprised of five main components, beginning with (a) a simple hexamer repeat of (CCCTCT)_n_ at the 5′ end, followed by (b) an *Alu*-like region made up of 2 antisense *Alu* fragments separated by a region of intervening sequence, (c) a variable number tandem repeat (VNTR), (d) a SINE region derived from the 3′ long terminal repeat (LTR) of the retroviral HERV-K10 element, and finally (e) a 3′ poly(A) signal [3] (Figure 1). A typical full length SVA element is several thousand bases in size, however, due to the young evolutionary age and the repetitive nature of the SVA structure, many SVA elements are polymorphic in the human population both in their structure and in their presence or absence. Structural polymorphism typically occurs within the (CCCTCT)_n_ hexamer repeat, the VNTR, and in the poly(A) region [11]. On the other hand, presence or absence polymorphisms (known as retrotransposon insertion polymorphisms (RIPs)) refer to sites at which the presence of an SVA is not yet fixed in the human population, and thus may be present at a particular locus in some individuals but absent in others.

In the case of the seventh SVA subfamily, known as F1, this group lacks the 5′ (CCCTCT)_n_ hexamer repeat, instead containing a 5′ transduction of exon 1 of the *MAST2* gene [19]. SVA elements do not encode the necessary proteins for mobilisation, but rather co-opt the proteins encoded by LINE-1 retrotransposons for this purpose [20,21]. Mobilisation of SVAs is known to be repressed by zinc finger proteins of the KRAB family [22].

In this report, we have extended our previous study [15] by analysing the distribution of SVAs per megabase across the human reference genome, and separating this analysis by evolutionary age. This allowed us to identify patterns of SVA insertions across primate history, and to highlight possible gene networks whose regulation or expression may have been influenced by such changes in genomic structure through evolution. We find that SVAs are over-represented primarily at zinc finger gene clusters across multiple chromosomes. ZNF zinc fingers are the largest family of transcription factors in the human genome [23]. This gene family has a complicated evolutionary history due to multiple rounds of duplication between species, which has resulted in significant diversity, as well as their organisation in distinct gene clusters across the human genome [24,25,26]. Similar to SVAs, zinc finger genes have been found to be over-represented on chromosome 19, and their gene clusters are over-represented for other transposable elements, including endogenous retroviruses (ERVs) and long terminal repeat (LTR) retrotransposons [27,28]. This has led to the proposal of co-evolution between transposable elements and the zinc finger gene family [27,28], which we here extend to the SVA class of primate-specific transposable elements.

## 2. Results

### 2.1. SVAs Cluster at Specific Zinc Finger Loci, Particularly on Chromosome 19

**Figure 2:**
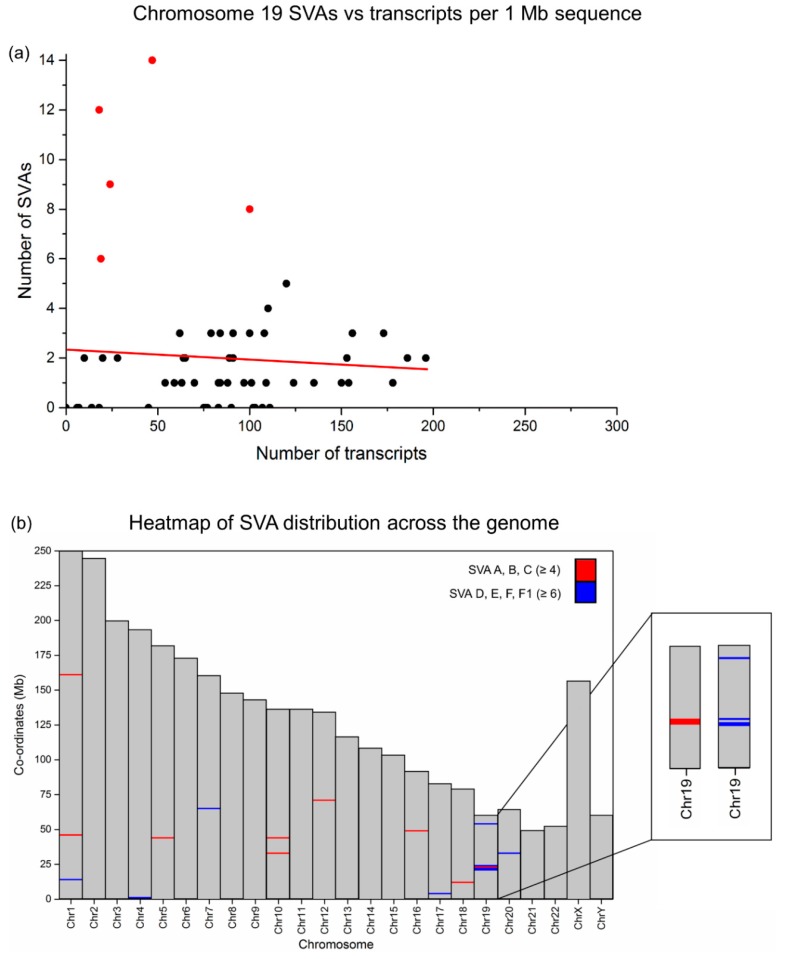
SVA elements preferentially cluster on chromosome 19. (**a**) Chromosome 19 is the only autosome that displays no correlation between SVA and transcript number per megabase (correlation coefficient = −0.055, Supplementary File 3). This apparent lack of correlation is skewed by a small number of regions which are vastly over-represented for SVAs based on their gene density (red points). (**b**) Mapping SVA density per megabase across the whole genome revealed regions with the highest number of SVAs. Red bars indicate regions with four or more SVA A, B, and Cs, while blue bars identify regions with six or more SVAs of the more recent D–F1 subfamilies. By overlaying SVA data for both older and younger SVA subclasses, we find that the chr19:20,000,000–24,000,000 locus is the only region in the genome at which clustering of both older and younger SVA classes is observed. This suggests sustained SVA-mediated evolution at this specific region on chromosome 19, from the evolution of the earliest SVA class to more recent human-specific changes. We further identify six regions that have high rates of SVA D–F1 subfamily insertions, including three additional zinc finger loci on chromosomes 4, 7, and 19.

To begin understanding the distribution of SVAs across the human genome through evolutionary time, the reference genome SVA set (hg19; Supplementary File 1) was analysed both in total and as two separate sets, one containing the evolutionary older subfamilies, SVA A, B, and C, and the other containing the more recent subfamilies D, E, F, and F1 (Supplementary File 2). We have previously demonstrated that SVAs are increased around genic regions at the genomic scale [11]. Here, we found that the trend towards increased SVA number with higher transcript number held true for 22 of the 24 chromosomes to varying degrees (Supplementary File 3). However, chromosome 19 and chromosome Y stood out clearly as the only chromosomes which deviated from this pattern, instead showing no correlation between SVA number and transcript number (correlation coefficients; Chr19 = −0.055 and ChrY = −0.140; Supplementary File 3). Since there are large regions of missing sequence data across Y chromosome on the UCSC Genome Browser, we were unable to determine whether this result was representative of the chromosome as a whole, and so did not continue further analysis. However, the lack of correlation on chromosome 19 appeared to be due to a small number of regions that were over-represented for SVAs in comparison to the number of transcripts at these regions (Figure 2a, red points). Overlaying the clustering patterns of the older SVA A, B, and C subfamilies with the more recent SVA D, E, F, and F1 subfamilies revealed that a four megabase stretch at Chr19:20,000,000–24,000,000 was the primary source of the lack of correlation seen between SVA number and transcript number on chromosome 19, and also represented the only region in the genome at which clustering of older and younger SVA subfamilies overlapped (Figure 2b).

**Figure 3:**
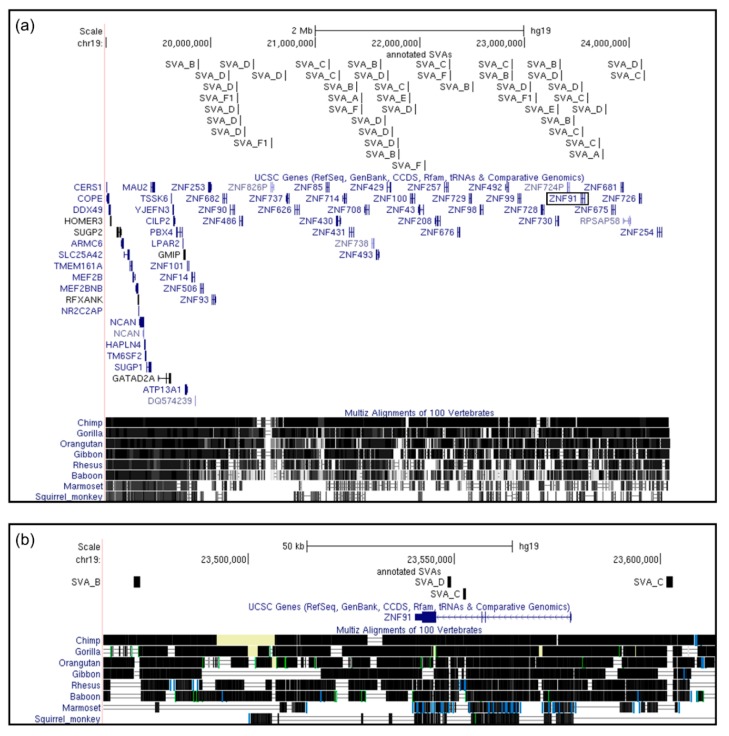
SVAs on chromosome 19 cluster at a KRAB ZNF zinc finger locus. (**a**) Visualisation of the chr19:20,000,000–24,000,000 locus and flanking regions (chr19:19,000,000–25,000,000; GRCh37/hg19) reveals that SVA clustering in this area falls directly across a large zinc finger gene locus, with no SVAs in the regions flanking either side. Observation of the Multiz vertebrate genome alignment data demonstrated significant change around this zinc finger locus through primate evolution, in clear contrast to the strongly conserved flanking region to the left. The white region on the right of the Multiz alignment track shows the centromere of chromosome 19, an area with no available sequence data. (**b**) The *ZNF91* gene resides in the chr19:20,000,000–24,000,000 SVA-rich zinc finger cluster (boxed in (**a**); chr19:23,464,801–23,613,281 shown in (**b**)), which is of interest due to its known role in suppressing SVA mobilisation. *ZNF91* is a primate-specific gene and contains an SVA C and SVA D insertion in the third intron which may have influenced expression or splicing of this gene specific to primate species from gorillas through to humans. Further, we observe a second SVA C approximately 24 kb upstream of the *ZNF91* transcriptional start site, and an SVA B around 67 kb downstream of the 3′ UTR. From these locations, all four SVAs have the potential to modulate *ZNF91* expression through methods including binding transcription factors, altering local chromatin structure, or modulating splicing.

Within this four megabase region, we identified 101 transcripts encoded by 47 genes, of which 32 (68.09%) were zinc finger genes (Figure 3a). Of the 32 zinc finger genes at this locus, 27 encoded zinc finger proteins that contained a KRAB domain. This is of interest as the KRAB domain-containing class of zinc finger proteins are known to bind and regulate the activity of retrotransposable elements [22]. Across this same region lay 41 SVAs (17 older SVAs, and 24 younger SVAs), which gave an average of one SVA per 2.46 transcripts, or one SVA per 1.14 genes.

**Figure 4:**
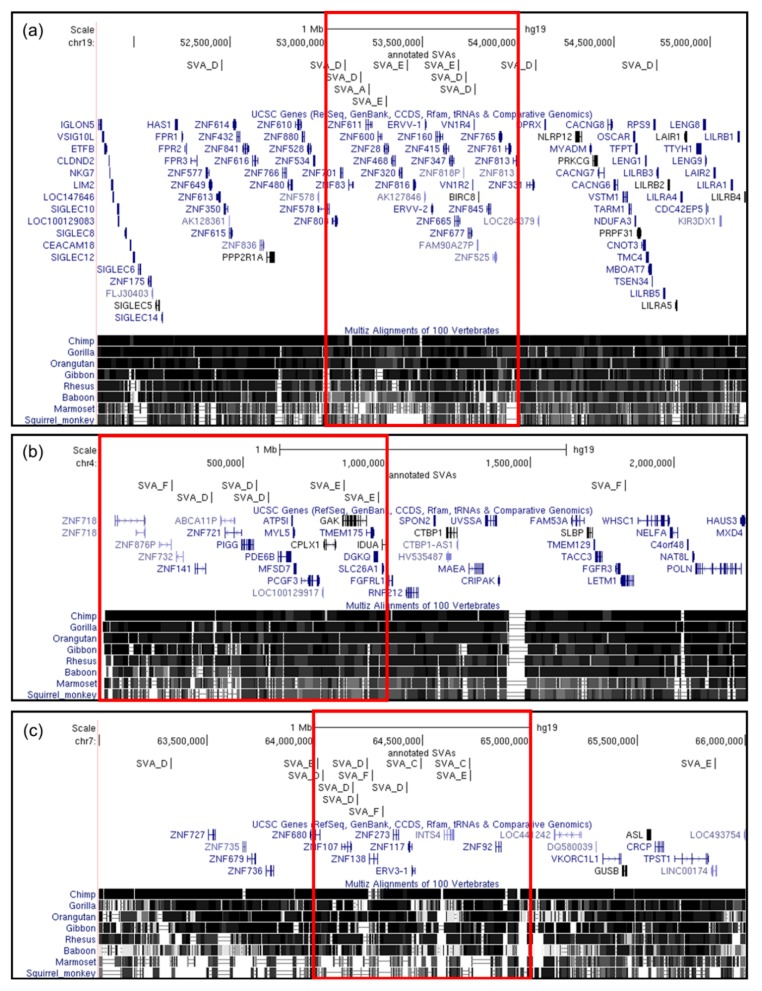
Three of the remaining five regions enriched for reference SVA D–F1 insertions are zinc finger clusters. In addition to the chr19:20,000,000–24,000,000 locus, three of the remaining five loci with the highest rates of SVA D–F1 insertions are at zinc finger regions. A second locus was identified on chromosome 19 at chr19:53,000,000–54,000,000, and two separate loci at chr4:1–1,000,000 and chr7:64,000,000–65,000,000 (expanded loci shown as chr19:51,812,498–55,187,502, chr4:1–2,250,000, and chr7:63,000,000–66,000,000). (**a**) The chr19:53,000,000–54,000,000 locus contains one SVA A, four SVA Ds, and three SVA Es, as well as 25 zinc finger genes. We observe the extension of this zinc finger gene cluster outside of this specific megabase, with additional SVA Ds present at either end of the zinc finger cluster. (**b**) A significantly smaller zinc finger gene cluster resides within the first megabase of chromosome 4 (chr4:1–1,000,000), encompassing three SVA Ds, two SVA Es, and an SVA F, arranged over a stretch of six zinc finger genes on the tip of the chromosome and into the remainder of this megabase. We note that the two human-specific SVA Es are directly over a genome-wide associated region for Parkinson’s disease (PD) encompassing the *GAK* and *DGKQ* genes, with one SVA E within a *GAK* intron, and the second lying within 2 kb of the *DGKQ* transcriptional start site. SVAs at this region could therefore modulate expression of both zinc finger and PD-related genes at this locus uniquely in humans. (**c**) The final SVA-rich zinc finger gene cluster is identified at chr7:64,000,000–65,000,000 and contains one SVA B, two SVA Cs, five SVA Ds, one SVA E, and two SVA Fs, spread across six zinc finger genes. Expanding this locus shows four additional zinc finger genes adjacent to this megabase, with a sixth SVA D.

The second smaller region highlighted on chromosome 19 (Chr19:53,000,000–54,000,000) was enriched only for the younger classes of SVAs (Figure 4a) and contained 33 genes (which encoded 100 transcripts), of which 25 (75.76%) were zinc finger genes of the ZNF family, with 20 of the 25 being KRAB domain-containing zinc finger genes. This one megabase locus contained eight SVAs, with an average of one SVA per 12.5 transcripts or per 4.13 genes. Of the remaining five loci at which clustering of a high number of younger SVAs was seen (Supplementary File 2), two of these, at Chr4:1–1,000,000 and at Chr7:64,000,000–65,000,000 were also zinc finger gene cluster regions (Figure 4b, c). Our data suggests a sustained drive for SVA-mediated evolution at the Chr19:20,000,000–24,000,000 locus throughout primate evolution over the last 13.6 million years, and a more recent evolution uniquely in higher primates, including chimpanzees and humans, involving multiple other zinc finger loci.

Three of the zinc finger clusters shown to be targeted by SVA insertions in this study (Chr19:20,000,000–24,000,000; Chr4:1–1,000,000; and Chr7:64,000,000–65,000,000) were part of the primate-specific *ZNF91* subfamily; a region which has been partially duplicated numerous times across multiple chromosomes throughout primate evolution [26].

The ZNF91 protein is known to repress SVA mobilisation [22], which is of interest given its location within the most SVA-dense region of the genome at the Chr19:20,000,000–24,000,000 locus. The region around the *ZNF91* gene has itself undergone SVA-mediated change, as was apparent from observation of the Multiz vertebrate sequence alignments that showed gaps in primate species conservation where SVAs have inserted at this locus. *ZNF91* had two chimp- and human-specific SVAs of the C and D classes within the third intron of the gene, an additional SVA C approximately 24 kb upstream of the *ZNF91* transcriptional start site, and an SVA B around 67 kb downstream of the 3′ UTR (Figure 3b). The location of these SVAs may impact *ZNF91* expression and splicing uniquely in higher primate species such as common chimpanzees and humans. It has previously been suggested that *ZNF91* underwent rapid evolutionary change around 8 to 12 million years ago [22], which coincided with the evolution and expansion of the SVA subfamilies B, C, and D, which are found at this gene, around 11.6, 10.9, and 9.6 million years ago [3].

**Figure 5:**
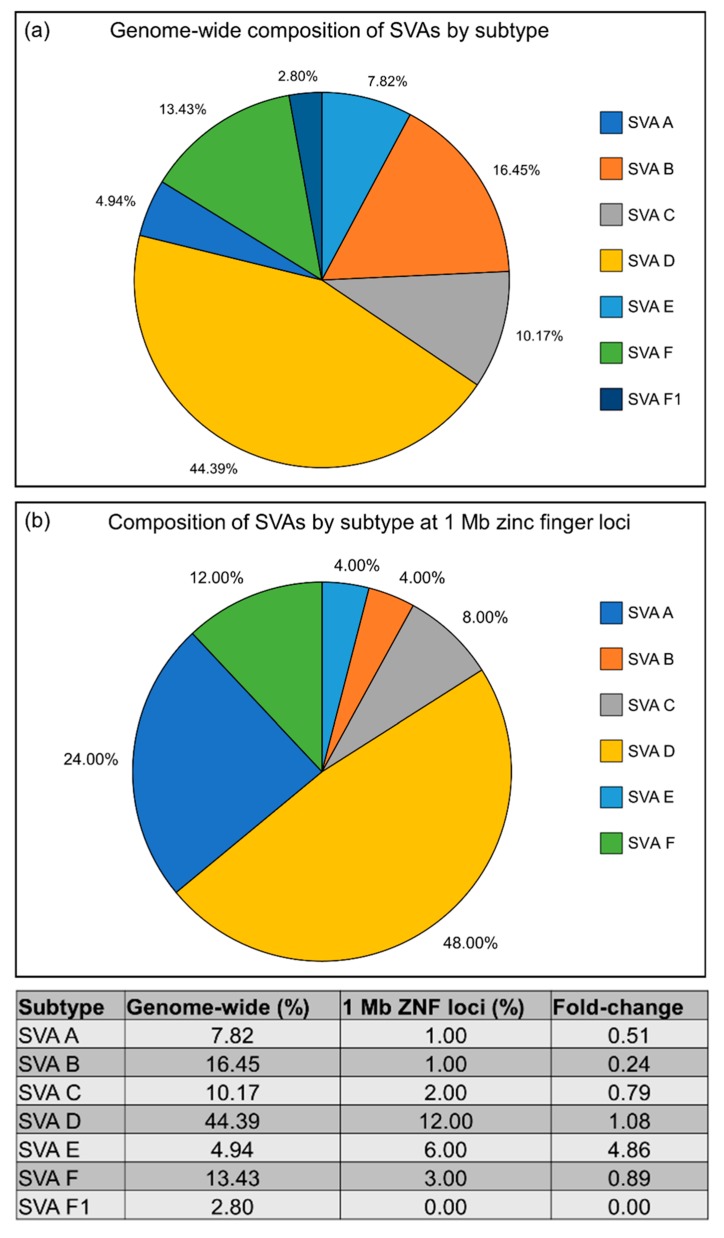
Composition of SVA subfamilies within 1 Mb zinc finger gene regions compared to the whole genome. (**a**) Calculating the percentage of each SVA subfamily across the human genome demonstrated that 34.44% of all SVAs were comprised of the evolutionary older A–C subfamilies and are therefore conserved at their respective loci within multiple primate species including humans. The remaining 65.56% is comprised of younger subfamily members D–F1. SVA D is by far the largest SVA subfamily, comprising 44.39% of all SVA elements in the genome, some of which are human-specific, with others being present in multiple primate species. 21.17% of SVAs (subfamilies E–F1) are entirely human-specific. SVA B, F, and C each represent over 10% of all SVAs, with a percentage of 16.45, 13.43, and 10.17%, respectively. The remaining subfamilies (SVA A, E, and F1) make up less than 10% of the total each. (**b**) The percentage of each SVA subfamily at the 1 Mb SVA clustered ZNF loci on chromosomes 4, 7, and 19 showed that older SVA subfamilies SVA A–C were reduced by 2.15-fold to 16.00% compared to the whole genome composition. This shift is due to an under-representation of SVA A and B subtypes (0.51- and 0.24-fold change, respectively), and a strong 4.86-fold increase in the SVA E subfamily = at the identified ZNF loci.

In order to more clearly understand the increase in young SVA subfamilies at the above identified 1 Mb zinc finger loci, the number of elements from each SVA subfamily at these loci were compared with SVA subfamily elements across the whole genome. Notable differences were identified as seen in Figure 5. Across the entire genome, older SVA subfamilies SVA A–C comprise 34.44% of all SVAs, while these subfamilies account for only 16.00% of SVAs identified at the 1 Mb zinc finger loci on chromosomes 4, 7, and 19 (0.46-fold change). This difference is predominantly driven by an under-representation of the oldest SVA subtypes A and B at zinc finger loci, and a strong over-representation of the SVA E subfamily. No SVA F1 elements were identified at the three zinc finger cluster regions used in this analysis (chr4:1–1,000,000, chr7:64,000,000–65,000,000, chr19:53,000,000–54,000,000), and thus we were not able to compare change in this subtype. Given that the SVA F1 subtype is the most recently evolved SVA element, and is the smallest in number across the genome, their lack of representation at the three zinc finger regions used in this analysis is perhaps not unexpected.

SVA A and B subfamilies were found to display a 0.51-fold and 0.24-fold change in occurrence at the 1 Mb zinc finger loci compared to the genome-wide average, while the SVA D and F subfamilies remained fairly constant with a 1.08 and 0.89-fold-fold change, respectively. On the other hand, the SVA E subfamily was over-represented by 4.86-fold (Figure 5). This is of particular interest given the increased GC content in human-specific SVA elements, and their increased potential to form G-quadruplex structures (via the (CCCTCT)_n_ hexamer repeat) that are known to modulate expression of neighbouring genes [11,17,29,30]. The SVA F1 subfamily was not present in any of the three ZNF loci in this analysis, but was over-represented at the 4 Mb loci on chromosome 19 compared to the genome-wide average (chr19:20,000,000–24,000,000; 3.15-fold change). Damert et al. have hypothesised that the 5′ transduction of the GC-rich *MAST2* exon 1, which has been incorporated into the SVA F1 structure, may confer an advantage to this SVA subfamily leading to increased copy number in the genome [31,32].

Work by Imbeault et al. used ChIP-exo (a high resolution modification of ChIP-seq) to profile the binding enrichment of 159 zinc finger proteins (ZFPs) across a wide range of different transposable elements [33]. We made use of this publicly available data set to assess KRAB-ZFPs that demonstrated significant binding enrichment at SVA elements. Of the 159 proteins profiled, 15 showed a significant enrichment for binding at one or more SVA subfamilies using the *p*-value cut-off of 1 × 10^−20^ (Table 1). Six of these 15 proteins (40%) were within the previously identified SVA-dense regions. Strikingly, we find that zinc finger proteins that are enriched for binding the older SVA subfamilies (SVA A–C) reside exclusively within regions identified as over-represented for SVAs in our above analysis. On the other hand, the nine ZFPs outside of SVA-dense regions that are enriched for SVA binding only demonstrate significance for their enrichment at younger SVA subfamily elements (SVA D–F). Notably, ZNF611 (residing within the chr19:53,000,000–54,000,000 locus) displays highly significant enrichment for binding across all SVA subfamilies, with a *p*-value of 1.14 × 10^−320^ for enrichment at SVA A–D and F elements, and 7.8 × 10^−200^ at SVA E elements.

### 2.2. Analysis of Human Retrotransposon Insertion Polymorphisms Suggests Continued Evolution of Zinc Finger Loci

SVAs remain mobile in the human genome, with recent studies suggesting multiple new and unique somatic insertions in the brain of each individual [34,35,36], as well as numerous unique germline insertions [37,38,39]. In order to extend this study to cover the patterns of continued SVA-mediated diversity in modern humans, we made use of the TEBreak tool (https://github.com/adamewing/tebreak) which contained a list of known and predicted germline SVA retrotransposon insertion presence/absence polymorphisms (RIPs), and repeated the above analysis for such RIPs across the genome per megabase (Supplementary File 2).

**Figure 6:**
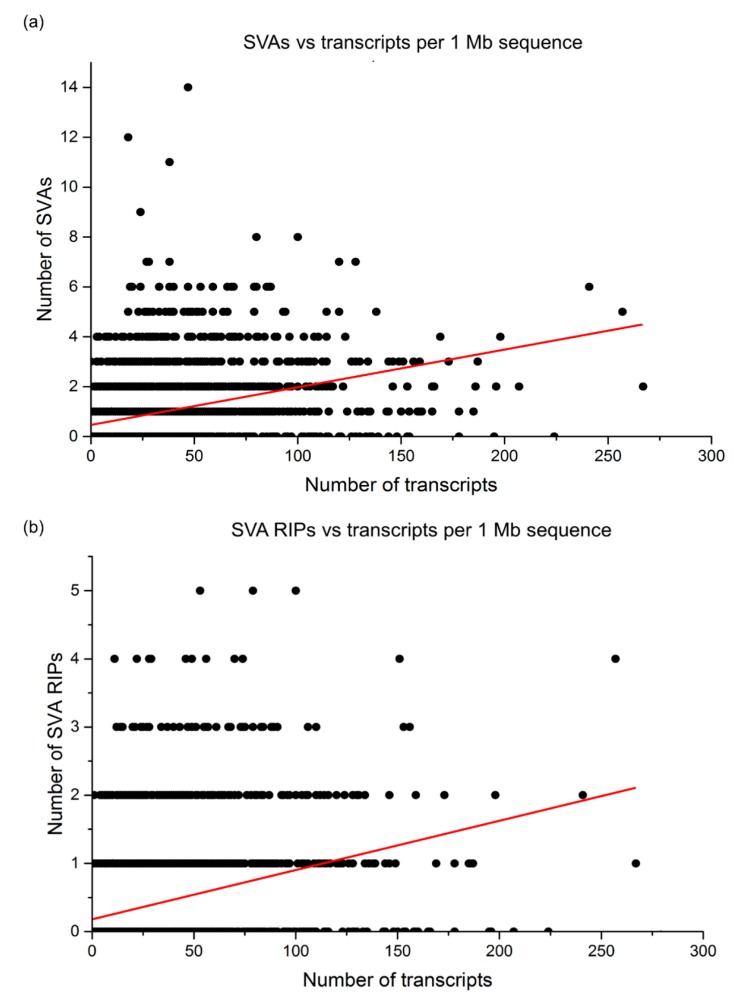
Both reference SVA insertions and SVA retrotransposon insertion polymorphisms (RIPs) are preferentially found at genic regions. (**a**) Plotting the number of reference SVAs vs. transcripts per megabase across the whole genome shows that SVAs are preferentially found at genic regions, with higher transcript number per megabase correlating with higher SVA number (correlation coefficient = 0.352, Supplementary File 3). (**b**) Plotting the number of SVA retrotransposon insertion polymorphisms (RIPs) vs. transcript number per megabase shows that new and polymorphic SVA insertions follow the same trend as established reference SVAs, with a preference for genic regions (correlation coefficient = 0.306, Supplementary File 3).

**Figure 7:**
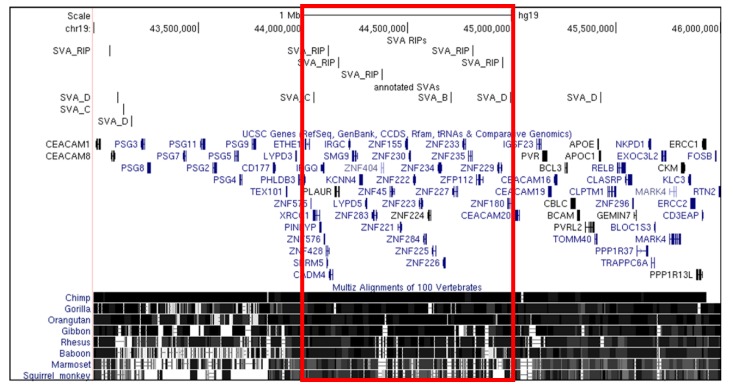
SVA retrotransposon insertion polymorphisms (RIPs) cluster at a third zinc finger gene locus on chromosome 19. Distribution analysis of SVA RIPs per megabase revealed three regions with the highest density of new and polymorphic SVA insertions in humans, with five SVA RIPs per megabase. Visualisation of these regions using the UCSC Genome Browser showed that the chr19:44,000,000–45,000,000 locus (chr19:43,000,000–46,000,000 shown above) contains an additional zinc finger gene cluster. With only three reference SVA insertions, this zinc finger cluster appears to be the target of SVA RIP insertion independently of previous reference SVA-mediated evolution, which we have shown to occur at other zinc finger gene loci. This suggests that SVA-mediated evolution of zinc finger clusters may be ongoing, with the chr19:44,000,000–45,000,000 locus in particular continuing to undergo genomic change which may modulate the expression of genes at this locus uniquely in modern humans, and differently between individuals based on the presence or absence of these insertions.

The list from TEBreak identified 1148 SVA RIPs, and distribution analysis demonstrated that these RIPs follow the same general trend as reference SVAs, with a positive correlation between transcript number and SVA RIP number (correlation coefficient = 0.31; Figure 6; Supplementary File 3). Distribution analysis revealed three loci with the highest rate of polymorphic SVA insertion, with five identified SVA RIPs each at Chr1:28,000,001–29,000,000, chr9:134,000,001–135,000,000, and Chr19:44,000,001–45,000,000. Of these three, the Chr19:44,000,001–45,000,000 locus was found to be another zinc finger cluster (Figure 7). This region had three reference SVAs and was therefore not considered to be over-represented for reference SVA insertions. This may suggest SVA-mediated evolution at a further zinc finger locus which is ongoing in modern humans.

## 3. Discussion

Our finding that human reference genome SVAs cluster primarily at zinc finger loci on chromosome 19 is in line with previous observations that this chromosome is enriched for transposable elements [3,12,13], and particularly that zinc finger gene clusters on this chromosome are over-represented for other transposable element classes such as ERVs and LTRs [27,28]. Taking a more detailed view across the genome by megabase highlighted an over-representation of SVAs at zinc finger gene clusters, both at multiple sites across chromosome 19, and additionally on chromosomes 4 and 7. We demonstrate that, outside of the Chr19:20,000,000–24,000,000 locus, this pattern largely involves younger SVA subfamilies, and observe suggestive evidence that this process may be ongoing in humans, with the distribution of polymorphic SVA insertions highlighting an additional chromosome 19 zinc finger locus with a high density of SVA RIPs. Taken together, these data may suggest that the movement of SVA subfamilies across higher primate and human genomes could have impacted a broad range of transcriptional networks through SVA targeting of zinc finger gene clusters. KRAB ZNFs and SVAs are known to have co-evolved, with the evolution and expansion of new SVA subfamilies across the genome prompting multiple rounds of zinc finger gene evolution in order to continue repressing the mobilisation of newly evolving SVA classes [22]. ZNF proteins have been shown to bind fragments of retrotransposon sequence and use these as platforms for the regulation of nearby genes [33]. In SVAs, the VNTR region was shown to be sufficient for ZNF91 binding and repression of SVA mobilisation [22]. It is known that ZNFs repress retrotransposons through the recruitment of TRIM28 and subsequent alteration of histone modifications. Studies of TRIM28-mediated repression of transposable elements, such as endogenous retroviruses, has demonstrated that histone modification at these elements affects local chromatin structure and regulation of neighbouring genes [40,41]. The over-representation of younger SVA subfamilies at the zinc finger loci identified here is also of interest due to the increased ability to regulate nearby gene expression. This heightened regulatory capacity of recently evolved SVA subfamilies is due to increased VNTR length and higher GC content, with the latter potentially forming the regulatory G-quadruplex DNA structure [11,17,31,32].

Given that zinc fingers are the largest class of transcription factors in the human genome [23,42], any SVA-mediated effects on their regulation could have the potential for wide-ranging transcriptional and epigenetic consequences. Data from the Geneotype-Tissue Expression project (GTEx; https://gtexportal.org) highlights many eQTLs residing in SVA sequences which are thought to be associated with zinc finger expression across a wide range of tissues. While interesting, it is difficult to disentangle linkage disequilibrium to identify the SNPs with truly causal effects on gene expression. However, such efforts may be of interest with regard to directing future research in this area. Further to this, work by Imbeault et al. identified 15 KRAB-ZFPs that are significantly enriched for binding at SVA elements. Six such ZFPs reside within the above identified SVA-dense loci, and intriguingly, ZFPs that bind older SVA subfamilies (SVA A–C) are restricted to these SVA-dense genomic loci (Table 1).

The process of retrotransposon mobilisation is ongoing in humans, and has been demonstrated to have impacts both in human health and disease. For example, X-Linked Dystonia-Parkinsonism (XDP), is a progressive neurodegenerative condition which is thought to be caused by a polymorphic SVA insertion in the 32nd intron of the *TAF1* gene [43]. Variation in the repeat length of the (CCCTCT)_n_ domain of this SVA in those with XDP has been significantly correlated with age of onset, symptom severity, and expression of the TAF1 gene in patient blood samples [44,45]. Further, a polymorphic SVA E insertion within intron 8 of the *CASP8* gene, tagged by the variant rs700635 (C), has been associated with *CASP8* splicing abnormalities, as well as increased risk of cutaneous basal cell carcinoma and breast cancer [46,47]. On the other hand, the same SVA has been associated with protection against prostate cancer [47], suggesting that such polymorphic insertions could play a variety of roles with regard to variation in human health and disease.

We identified 1148 polymorphic SVA insertions in the human population, which are present or absent in the genome, and demonstrated that these elements followed similar insertion patterns to their reference genome counterparts. SVA RIPs were found at increased levels in genic regions, and particularly at an additional zinc finger gene locus on chromosome 19, suggesting the potential for continued SVA-mediated evolution of zinc finger gene loci in humans.

Taken together, we observe that SVAs have continually inserted and been retained at ZNF loci, and may be continuing to do so in modern human populations. Work by Jacobs et al. has provided evidence of SVA and zinc finger co-evolution [22], thereby supplying one potential mechanism driving retrotransposon-mediated evolution of this large class of transcription factors in higher primates and humans. SVAs are known to be bound by a number of KRAB zinc finger proteins [33], as well as regulators of chromatin architecture including CTCF and CTCFL [48]. Such retrotransposon-mediated changes in genomic structure may therefore play a role in the 3D organisation of chromatin, with the potential to affect gene expression patterns more globally, though future research efforts will be required in order to test this hypothesis.

## 4. Methods

### 4.1. Co-Ordinates of Reference Genome and Insertion Polymorphism SVA Elements

Annotations for reference genome SVA elements were accessed through the UCSC table browser using the ‘repeat masker’ track from human genome build 19 (GRCh37/hg19), and were subsequently corrected for overlapping or split reads.

The list of retrotransposon insertion polymorphisms was compiled by Adam Ewing and is available through the TEBreak programme, which characterises non-reference retrotransposon insertions (https://github.com/adamewing/tebreak). This list was compiled using known or predicted non-reference germline insertions found in multiple studies assessing RIPs in humans [37,38,39,49,50,51].

### 4.2. Transcript Data and SVA Distribution Analysis

The number of retrotransposon insertions per megabase was counted using custom scripts written in R, and the number of transcripts per megabase was accessed through R using the ‘TxDb.Hsapiens.UCSC.hg19.knownGene’ library via a script written by Dr. Giovanni M Dall’Olio and published through the BioStars website (https://www.biostars.org/p/169171/#169211).

## Figures and Tables

**Table 1:** Publicly available KRAB-zinc finger protein (ZFP) binding enrichment data from ChIP-exo studies by Imbeault et al. [33]. Fifteen ZFPs were found to be significantly enriched for binding one or more SVA subfamilies at the *p*-value cut-off of 1 × 10^−20^. Six such ZFPs were located at regions found to be over-represented for SVAs. Notably, ZFPs displaying enrichment for older SVA subfamilies (SVA A–C) were only found within SVA-rich zinc finger loci.

Protein	Position	SVA Locus	Binding Enrichment (*p*-Value)					
			SVA A	SVA B	SVA C	SVA D	SVA E	SVA F
ZNF141	chr4:331,596–367,691	**Y**	7.76 × 10^−6^	***4.53 × 10^−89^***	***1.04 × 10^−62^***	5.07 × 10^−5^	0.13	0.40
ZNF736	chr7:63,773,186–63,810,017	**Y**	0.61	1.00	0.53	3.13 × 10^−14^	3.98 × 10^−4^	***1.24 × 10^−23^***
ZNF257	chr19:22,235,266–22,273,903	**Y**	***4.43 × 10^−83^***	4.11 × 10^−10^	4.85 × 10^−4^	2.11 × 10^−6^	0.19	4.9 × 10^−5^
ZNF730	chr19:23,299,777–23,330,017	**Y**	1.00	1.00	0.17	***2.97 × 10^−27^***	6.05 × 10^−7^	***3.99 × 10^−42^***
ZNF611	chr19:53,206,066–53,238,307	**Y**	***1.14 × 10^−320^***	***1.14 × 10^−320^***	***1.14 × 10^−320^***	***1.14 × 10^−320^***	***7.8 × 10^−200^***	***1.14 × 10^−320^***
ZNF28	chr19:53,300,661–53,324,922	**Y**	***1.09 × 10^−67^***	***1.09 × 10^−22^***	***4.62 × 10^−25^***	***2.5 × 10^−234^***	3.37 × 10^−4^	***3.2 × 10^−110^***
ZKSCAN5	chr7:99,102,273–99,131,445	N	2.93 × 10^−13^	9.43 × 10^−5^	8.21 × 10^−3^	***2.6 × 10^−39^***	***2.77 × 10^−31^***	***1.92 × 10^−74^***
ZNF202	chr11:123,594,997–123,612,363	N	0.91	0.88	0.73	2.52 × 10^−12^	2.04 × 10^−13^	***7.67 × 10^−57^***
ZNF641	chr12:48,733,793–48,744,674	N	0.01	1.71 × 10^−5^	3.79 × 10^−13^	***2.7 × 10^−201^***	***4.1 × 10^−104^***	***2.4 × 10^−304^***
ZNF605	chr12:133,498,019–133,532,892	N	7.96 × 10^−3^	0.07	0.04	***7.83 × 10^−58^***	***1.03 × 10^−23^***	***4.68 × 10^−67^***
ZNF558	chr19:8,920,382–8,933,565	N	0.05	1.00	1.69 × 10^−4^	***8.94 × 10^−57^***	***1.55 × 10^−30^***	***6.4 × 10^−135^***
ZNF30	chr19:35,417,807–35,436,076	N	1.00	0.14	2.22 × 10^−4^	***2.56 × 10^−63^***	***5.32 × 10^−28^***	***2.5 × 10^−134^***
ZNF780A	chr19:40,578,899–40,596,845	N	0.25	3.70 × 10^−3^	1.79 × 10^−3^	***1.14 × 10^−26^***	0.02	2.59 × 10^−14^
ZNF649	chr19:52,392,488–52,408,305	N	1.87 × 10^−3^	0.06	7.23 × 10^−6^	***6.1 × 10^−158^***	***9.68 × 10^−66^***	***3.9 × 10^−191^***
ZNF334	chr20:45,128,269–45,142,198	N	1.00	0.05	1.00	2.1 × 10^−12^	2.91 × 10^−17^	***2.14 × 10^−21^***

## Supplementary Materials

Supplementary materials can be found at https://www.mdpi.com/1422-0067/20/23/5977/s1. Supplementary File 1—Excel spreadsheet detailing the co-ordinates of all SVA elements in the human reference genome (hg19). Supplementary File 2—Excel spreadsheet detailing the number transcripts, reference genome SVAs, and SVA insertion polymorphisms (RIPs) per megabase of the human genome (hg19). Supplementary File 3—Excel spreadsheet of correlation coefficients for transcript number and SVA number for each human chromosome.
